# Epigenetic Regulation of Pluripotent Genes Mediates Stem Cell Features in Human Hepatocellular Carcinoma and Cancer Cell Lines

**DOI:** 10.1371/journal.pone.0072435

**Published:** 2013-09-04

**Authors:** Xiao Qi Wang, Ray Kit Ng, Xiaoyan Ming, Wu Zhang, Lin Chen, Andrew C. Y. Chu, Roberta Pang, Chung Mau Lo, Sai Wah Tsao, Xuqing Liu, Ronnie T. P. Poon, Sheung Tat Fan

**Affiliations:** 1 Department of Surgery, The University of Hong Kong, Pokfulam, Hong Kong, China; 2 State Key Laboratory for Liver Research, The University of Hong Kong, Pokfulam, Hong Kong, China; 3 Department of Pathology, The University of Hong Kong, Pokfulam, Hong Kong, China; 4 Center for Reproduction, Development and Growth, The University of Hong Kong, Pokfulam, Hong Kong, China; 5 Center for Cancer Research, The University of Hong Kong, Pokfulam, Hong Kong, China; 6 Department of Anatomy, The University of Hong Kong, Pokfulam, Hong Kong, China; University of Delhi, India

## Abstract

Activation of the stem cell transcriptional circuitry is an important event in cancer development. Although cancer cells demonstrate a stem cell-like gene expression signature, the epigenetic regulation of pluripotency-associated genes in cancers remains poorly understood. In this study, we characterized the epigenetic regulation of the pluripotency-associated genes *NANOG*, *OCT4*, *c-MYC*, *KLF4*, and *SOX2* in a variety of cancer cell lines and in primary tumor samples, and investigated the re-activation of pluripotency regulatory circuits in cancer progression. Differential patterns of DNA methylation, histone modifications, and gene expression of pluripotent genes were demonstrated in different types of cancers, which may reflect their tissue origins. *NANOG* promoter hypomethylation and gene upregulation were found in metastatic human liver cancer cells and human hepatocellular carcinoma (HCC) primary tumor tissues. The upregulation of *NANOG*, together with p53 depletion, was significantly associated with clinical late stage of HCC. A pro-metastatic role of NANOG in colon cancer cells was also demonstrated, using a *NANOG*-overexpressing orthotopic tumor implantation mouse model. Demethylation of *NANOG* promoter was observed in CD133+^high^ cancer cells. In accordance, overexpression of *NANOG* resulted in an increase in the population of CD133+^high^ cells. In addition, we demonstrated a cross-regulation between *OCT4* and *NANOG* in cancer cells via reprogramming of promoter methylation. Taken together, epigenetic reprogramming of *NANOG* can lead to the acquisition of stem cell-like properties. These results underscore the restoration of pluripotency circuits in cancer cells as a potential mechanism for cancer progression.

## Introduction

Epigenetic changes are considered as potent surrogates to mutations in the deregulation of growth-promoting genes and tumor-suppressor genes [1], [2]. It has been proposed that the process of carcinogenesis involves epigenetic alterations in stem/progenitor cells before gatekeeper gene mutations occur [1], [3], [4]. This process can presumably affect both the genetic and epigenetic plasticity of a cell and allow acquisition of “stemness” features, such as invasion, metastasis and drug resistance, during cancer progression [1], [2]. Moreover, genomic instability caused by global DNA hypomethylation was found to be one of the earliest changes in the development of human cancers [2], [5].

While overexpression of *OCT4*, *SOX2*, *KLF4* and *c-MYC* genes induce pluripotency in somatic cells leading to the generation of embryonic stem cell (ESC)-like induced pluripotent stem cells (iPSCs) [6]–[8], it is interesting to note that the ESC-like transcriptional program is often activated in diverse human epithelial cancers [9], [10]. Such an ESC-like gene module was also associated with disease progression, e.g. metastasis, and early mortality of breast cancer [9] and bladder cancer [11]. It, therefore, suggests a common molecular pathway involved in both iPSC derivation and cancer stem cell (CSC) initiation [12]. Moreover, recent studies have demonstrated that iPSCs retain epigenetic memory, such as DNA methylation signature, from their tissue origins [13], [14], indicating the importance of epigenetic regulation in cell fate reprogramming and tumorigenesis [15], [16].

Although stem cell-like gene network has been demonstrated in cancers [11], the association of epigenetic reprogramming and CSC properties remains poorly understood. Here, we investigated the epigenetic regulation of pluripotency-associated genes *NANOG*, *OCT4*, *c-MYC*, *KLF4*, and *SOX2*, and their correlation with gene expression in cancer cell lines and primary tumor samples. We further examined their potential roles in the process of metastasis and in the initiation of CSCs during tumor progression.

## Materials and Methods

### Samples

Paired non-tumor and tumor tissue specimens of hepatocellular carcinoma (HCC) were collected from fifteen HCC patients diagnosed with stage I-IV pathologic tumor-node-metastasis (TNM) disease [17]. Normal liver specimens were obtained from cadaveric liver donors. All the samples were provided by the Tissue Bank of Divisions of Hepatobiliary and Pancreatic Surgery and Liver Transplantation at Department of Surgery, Queen Mary Hospital. Collection and storage of clinical specimens for the Tissue Bank has been approved by the Institutional Review Board of the University of Hong Kong/Hospital Authority of Hong Kong. Normal hepatocyte and HCC cell lines included MIHA and L02 (normal hepatocytes); PLC (primary HCC); and MHCC97L (97L) and MHCC97H (97H), were derived from metastatic HCC [18]. Other non-HCC cancer cell lines included HeLa (cervix adenocarcinoma); MCF7 (breast adenocarcinoma); AGS (gastric adenocarcinoma); HCT116 (colorectal carcinoma); and K-562 (chronic myelogenous leukemia). The PLC, HeLa, MCF7, AGS, and HCT116 lines were purchased from the American Type Culture Collection (ATCC). L02 was obtained from the China Center for Type Culture Collection.

### Genomic DNA isolation

Total genomic DNA was isolated from peripheral blood mononuclear cells (PBMC), cell lines, and non-tumor and tumor liver specimens from HCC patients, using the QIAamp DNA mini kit (Qiagen).

### Bisulfite sequencing analysis

**Figure 1:**
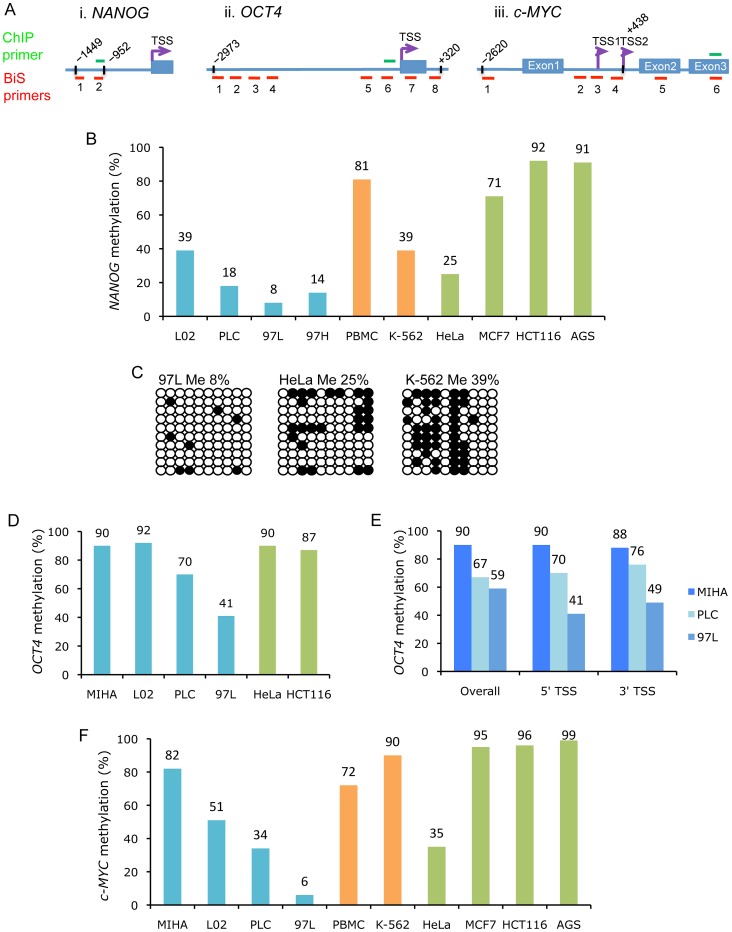
Differential methylation of pluripotency-associated genes *NANOG*, *OCT4*, and *c*-*MYC* in cancer cells. (A) Schematic diagram of gene regulatory regions of *NANOG*, *OCT4*, and *c-MYC* that were examined by bisulfite sequencing (BiS) (red bars) and ChIP (green bars) experiments. (i) The *NANOG* proximal promoter region covers 10 CpG sites from −1449 to −952. (ii) The *OCT4* promoter region is covered by 8 primer pairs for 50 CpG sites from −2973 to +320. (iii) The *c-MYC* gene region is covered by BiS primers for CpG islands before TSS1 and TSS2, and CpG sites within Exon 2 and 3; and ChIP primer for Exon 3. (B) Bisulfite sequencing analysis of the *NANOG* promoter in cancer cell lines. DNA methylation frequency is presented as percentages in: normal liver (L02) and cancer liver (PLC, 97L, and 97H) cells (blue); normal PBMC and leukemic K-562 cells (orange); and in HeLa, MCF7, HCT116, and AGS cancer cells (green). (C) Clonal *NANOG* promoter methylation patterns in 97L, HeLa and K-562 cells. Open circles represent unmethylated CpGs; closed circles represent methylated CpGs. (D) Methylation frequency of the *OCT4* proximal promoter (−530 to +7) in: normal and cancer liver cells (blue); and in HeLa and HCT116 cells (green). (E) DNA methylation frequency of upstream and downstream regions of *OCT4* in normal and cancer liver cells. “Overall” covers 50 CpG sites from −2973 to +320; “5′ TSS” covers 10 CpG sites from −530 to +7; and “3′ TSS” covers 12 CpG sites from +61 to +320. (F) Methylation frequency of exon 3 of *c-MYC* (10 CpG sites) in: normal and cancer liver cells (blue); PBMC and leukemic K-562 cells (orange); and in HeLa, MCF7, HCT116, and AGS cancer cells (green).

Genomic DNA was processed for bisulfite conversion of unmethylated cytosines using the EpiTect Bisulfite kit (Qiagen). The bisulfite-modified DNA was used for PCR, with primers that recognize the converted DNA sequences (Figure 1A; Figure S1 in File S1; Table S1). PCR products were cloned into pGEM T-Easy vector (Promega Bioscience). Ten clones were randomly picked and sequenced. DNA sequences were analyzed with the BIO Analyzer software (http://biq-analyzer.bioinf.mpi-sb.mpg.de) for CpG reading. Percentage of methylation refers to the number of methylated CpGs over the total number of CpGs in the region analyzed.

### Chromatin immunoprecipitation (ChIP)

The ChIP procedure was described in Current Protocols [19]. Briefly, ten to twenty million (1–2×10^7^) L02, HCT116 and 97L cells were collected and fixed with 37% formaldehyde. The sonicated cell lysates were subjected to immunoprecipitation with 5 μg of H3K4me3 or H3K27me3 antibodies (Abcam), and normal IgG (inputs as control), respectively, in the presence of protein G-Sepharose beads (GE Healthcare) at 4°C overnight. Beads were washed sequentially with FA lysis buffer, ChIP wash buffer, and TE buffer. Bound materials were eluted with ChIP elution buffer. Enrichment of histone modification was quantified by real time PCR, using SYBR Green (Applied Biosystems), with relevant ChIP primers (Figure 1A; Figure S1 in File S1; Table S1). Data are presented as fold enrichment, using a ChIP-qPCR analysis calculation formula (www.sabiosciences.com); inputs and mock IP were used for normalization.

### Reverse transcription and quantitative real time PCR (qRT-PCR)

Total RNA was isolated using the RNeasy Mini kit (Qiagen) and treated with DNase I, followed by reverse transcription with the Transcriptor First Strand cDNA Synthesis Kit (Roche). qRT-PCR analysis was performed using the pre-designed TaqMan probes for *NANOG* (Hs02387400_g1), *POU5F1* (Hs00999634_gH), *MYC* (Hs00153408_m1), and *18S* rRNA (4333760-0807027), which were selected from TaqMan Gene Expression Assays (Applied Biosystems). SYBR Green reagents (Applied Biosystems or TaKaRa Biotechnology) were used for detection of *OCT4*, *MYC*, *KLF4, p53* and *β-ACTIN* gene expression (primer information in Table S2). Gene expression was calculated as CT and normalized to the level of *18S* or *β-ACTIN*.

### Lentivirus transduction

Human *NANOG* (NM_024865) and *OCT4* (NM_002701) genes from human embryonic stem cell line were cloned into pWPI vector and were transfected with pCMV-dR8.91 and pMD2.G plasmids into the 293T packaging cell line. Viral supernatants were harvested 48 hours after transfection and precipitated using PEG-it Virus Precipitation Solution (System Biosciences). HCT116 p53−/− cells were infected with lentivirus expressing either *OCT4* or *NANOG* in the presence of 8 μg/ml protamine sulfate. Cell sorting was carried out using FACSAria (BD Biosciences) for isolation of transduced HCT116 p53−/− cells.

### In vivo metastasis assay by orthotopic colon tumor implantation

Four to six weeks old BALB/cAnN-nu (Nude) mice were obtained and maintained at Laboratory Animal Unit of The University of Hong Kong. All mouse experiments were approved by The Committee on the Use of Live Animals of The University of Hong Kong. Lentivirus infected HCT116 p53−/− cells were injected subcutaneously into nude mice to generate primary tumors. The primary tumors were dissected into 1–2 mm^3^ cubes and then implanted into the cecum of other nude mice [20]. Colon to lung tumor metastasis was examined after 4 weeks of cecum implantation. The whole lungs from nude mice were sectioned and stained with H&E. The colon tumor lesions in the lungs were examined by microscopy.

### Flow cytometry and cell sorting

CD133-PE (Miltenyi Biotec) labeled HCT116 cells were analyzed using FACSCalibur (BD Biosciences). CD133+^low^, CD133+^high^, and CD133− cells were subjected to cell sorting by FACSAria (BD Biosciences).

### Statistical analysis

Data were presented as the mean ± SD and the Student's *t* test was performed using SPSS software. *P*<0.05 was regarded as statistically significant.

## Results

### Differential DNA methylation of pluripotency-associated genes in cancer cells

Previous studies have demonstrated the activation of downstream targets of *NANOG*, *OCT4*, *SOX2* and *MYC* genes in aggressively growing human cancers [11]. We therefore investigated the CpG methylation pattern of *NANOG* (also known as *NANOG1*), *OCT4*, *SOX2*, *KLF4* and *c-MYC* in human cancer cell lines by bisulfite sequencing approach (Figure 1A; Figure S1 in File S1). Compared to normal liver cells L02, which displayed a modest methylation level (39%), DNA hypomethylation of *NANOG* (Figure 1A–i) was observed in three liver cancer cell lines, namely PLC, 97L and 97H (18%, 8% and 14%, respectively), among which the metastatic liver cell line 97L showed nearly absence of DNA methylation (Figure 1B and 1C). While in leukemic K-562 cells, *NANOG* methylation (39%) showed a 2-fold decrease when compared to normal peripheral blood mononuclear cells (PBMCs) (81%; Figure 1B and 1C). Cancer cells from other tissue origins displayed differential *NANOG* promoter methylation; for example, hypomethylation was observed in HeLa (25%), whereas hypermethylation was shown in MCF7, HCT116 and AGS (71%, 92% and 91%, respectively) (Figure 1B and 1C).

We next examined the *OCT4* promoter methylation pattern in cancer cells. The proximal promoter region (−503 to +7; Figure 1A–ii) of *OCT4* was found hypermethylated in L02 (92%), MIHA (90%), HeLa (90%) and HCT116 (87%) cells, which is in contrast to the reduced levels observed in PLC and 97L cells (70% and 41%, respectively; Figure 1D). We extended the methylation analysis to a total of 50 CpGs that cover both the distal promoter and downstream region of the transcription start site (TSS, −2973 to +320; Figure 1A–ii). The reduced methylation pattern was conserved at the analyzed *OCT4* gene region in PLC and 97L cells (Figure 1E). For the *c-MYC* gene, although the CpGs located upstream of TSS1 and TSS2 and within exon 2 remained unmethylated in both normal and cancer cells (Figure 1A–iii; Figure S2 in File S1), the methylation level of exon 3 was dramatically reduced and became hypomethylated in two liver cancer cell lines, PLC (34%) and 97L (6%), when compared to the moderate to high level of methylation (51% to 82%) in normal liver cells (Figure 1F). In contrast, cancer cells from other tissue origins, with the exception of HeLa, displayed hypermethylation status of *c-MYC* exon 3 (ranging from 72% to 99%; Figure 1F). On the other hand, CpG islands at the promoter regions of *KLF4* and *SOX2* genes remained unmethylated in all the examined cell lines (Figures S1, S3, S4 in File S1). Taken together, differential methylation pattern of pluripotency-associated genes was observed in human cancer cell lines from different tissue origins.

### Epigenetic regulations of pluripotent gene expression

**Figure 2:**
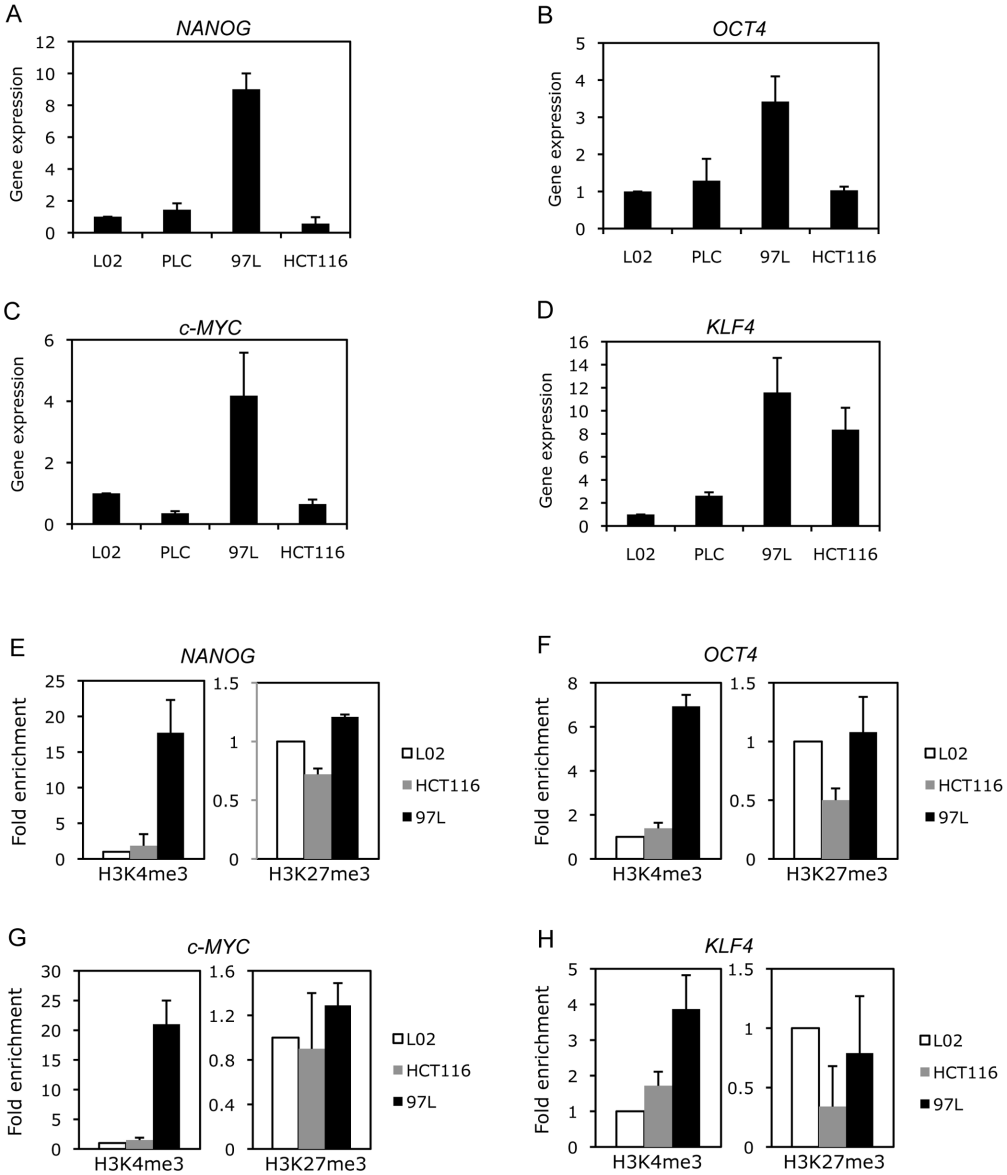
A correlation between pluripotency-associated gene expression and histone modification patterns in normal and cancer cells. Expression levels of (A) *NANOG*, (B) *OCT4*, (C) *c*-*MYC*, and (D) *KLF4* genes were determined in L02, PLC, 97L, and HCT116 cells by qRT-PCR analysis, normalized with the reference gene *18S*. The gene expression was normalized to L02 sample, which was defined as 1. Data are the mean ± SD obtained from 2 to 3 experiments with duplicates. Enrichment of histone modification H3K4me3 and H3K27me3 at the promoter regions of pluripotency-associated genes (E) *NANOG*, (F) *OCT4*, (G) *c*-*MYC*, and (H) *KLF4* were measured in L02, 97L, and HCT116 by ChIP analysis. Data are represented as fold enrichment and normalized with input and mock IgG controls. The fold enrichment was relative to L02 sample, which was defined as 1. Data are represented with mean value obtained from two ChIP experiments with error bars of standard derivation.

We next focused on the pluripotent gene expression in two cancer cell lines, 97L and HCT116, which showed opposite patterns of DNA methylation. qRT-PCR analysis demonstrated higher expression of *NANOG*, *OCT4* and *c-MYC* genes in 97L cells but not in HCT116 cells, when compared to control L02 and PLC cells (Figure 2A–C; Figure S5A–C in File S1), suggesting the expression of these genes is negatively regulated by DNA methylation. For *KLF4*, increased gene expression was observed in both 97L and HCT116 cells (Figure 2D; Figure S5D in File S1) despite the fact that the CpG island methylation pattern was indistinguishable between normal and cancer cells (Figure S3 in File S1).

Epigenetic modifications on histone proteins also showed strong association with gene expression. We, therefore, determined the presence of two kinds of histone modifications, the active/permissive tri-methyl-H3K4 (H3K4me3) and the repressive tri-methyl-H3K27 (H3K27me3), in 97L and HCT116 cancer cells. ChIP analysis demonstrated a significant enrichment of active H3K4me3 mark at the promoter regions of *NANOG* and *OCT4* and the exon 3 region of *c-MYC* in 97L cells, when compared to L02 and HCT116 cells (Figure 2E–G, left panels). This is in contrast to the low level of repressive H3K27me3 mark observed in these cells (Figure 2E–G, right panels). These results suggest that both histone modifications and DNA methylation function synergistically in regulating *NANOG*, *OCT4* and *c-MYC* gene expression. On the other hand, H3K4me3 was found highly enriched at the *KLF4* promoter of 97L cells, whereas HCT116 cells demonstrated a moderate level of H3K4me3 but with relatively lower level of repressive H3K27me3 (Figure 2H, right panel). This may account for the high level of *KLF4* gene expression in both cell lines (Figure 2D), which is independent of DNA methylation.

### NANOG promoter hypomethylation in human HCC tumor tissue

**Figure 3:**
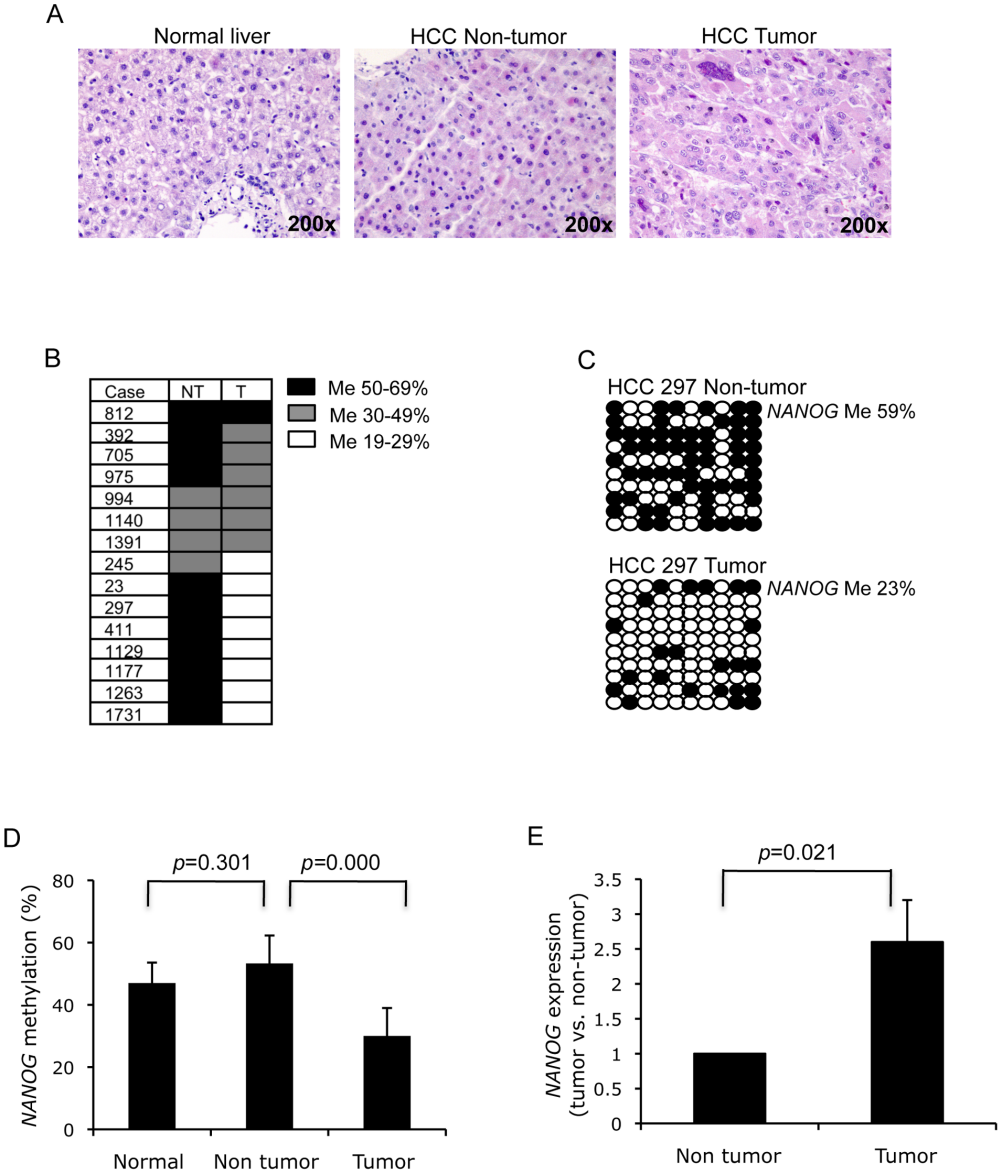
Deregulation of *NANOG* in HCC primary tumor tissue. (A) H&E staining of human normal liver (left), and HCC non-tumor (middle) and tumor tissue (right). (B) Methylation status of the *NANOG* promoter (−1449 to −952) in fifteen paired HCC non-tumor and tumor tissues. DNA methylation frequency was classified into 50–69% (black), 30–49% (grey), and 19–29% (white) in HCC tumor and adjacent non-tumor tissues. (C) *NANOG* promoter methylation pattern is represented with HCC case-297 tumor and adjacent non-tumor tissue. (D) Statistical comparison of *NANOG* promoter methylation in normal liver (n = 3), HCC adjacent non-tumor (n = 15) and tumor (n = 15) tissues; data are the mean ± SD. (E) Statistical comparison of *NANOG* gene expression in paired HCC non-tumor and tumor tissue (n = 15). Each tumor tissue was normalized with its corresponding non-tumor tissue.

Given that the differential *NANOG* methylation pattern in cancer cell lines can be a consequence of *in vitro* culturing rather than association with their tumorigenicity *per se*, we, therefore, investigated *NANOG* promoter methylation in primary HCC tumors paired with adjacent non-tumor tissues (n = 15), compared to normal liver samples (Figure 3A). Strikingly, 73% of non-tumor cases (11 out of 15) were more than 50% methylated, whereas 53% of tumor cases (8 out of 15) were less than 30% methylated at the *NANOG* promoter (Figure 3B and 3C). *NANOG* promoter methylation levels were significantly reduced in the paired HCC tumors compared with adjacent non-tumor tissues (*p* = 0.000), whereas no significant changes were detected between normal and non-tumor liver tissues (*p* = 0.301; Figure 3D). In accordance with the reduced promoter methylation, *NANOG* expression was found significantly higher in tumor tissue than in non-tumor tissue (*p* = 0.021; Figure 3E). It is worth noting that HCC samples at TNM stage III and IV, which is usually present with vascular invasion, lymph node and distant metastasis [17], was significantly associated with high *NANOG* (*p* = 0.011) and low *p53* (*p* = 0.047) expression (Table 1; Table S3). The low level of *p53* was also associated with vascular infiltration (*p* = 0.019; Table 1). These results suggest that tumor metastasis requires both the upregulation of *NANOG* via promoter hypomethylation and the suppression of *p53* in HCC.

**Table 1:** Correlation between metastatic clinicopathological parameters and mRNA expression of *NANOG* and *p53* in HCC.

Parameters	Category	*NANOG* mRNA		*p*	*p53* mRNA		*p*
		low	high		high	low	
Tumor TNM stage	I and II	5	1	0.011*	4	2	0.047*
	III and IV	1	8		1	8	
Venous infiltration	Absent	5	3	0.084	5	3	0.019*
	Present	1	6		0	7	
Tumor nodules	1 nodule	5	5	0.294	5	5	0.084
	≥2 nodules	1	4		0	5	
Tumor size	≤5cm	5	5	0.294	4	6	0.434
	>5cm	1	5		1	4	

* *p*<0.05 was considered as statistically significant.

### NANOG expression promotes tumor metastasis

**Figure 4:**
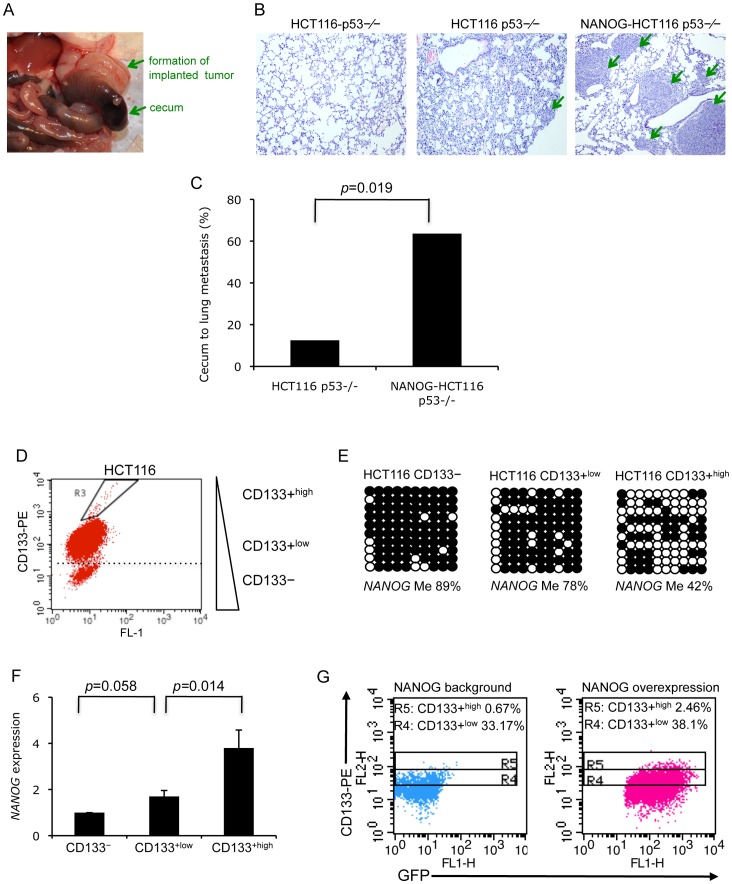
*NANOG* promotes tumor metastasis in a mouse model and enhances cancer cell CD133+^high^ population. (A) HCT116 p53−/− or NANOG-expressing HCT116 p53−/− cells (1×10^6^) were injected into nude mice subcutaneously. Xenograft tumor tissue that formed was excised and dissected into 1–2 mm^3^ cubes that were then implanted into the cecums of other nude mice. Tumor formation in the cecum was observed after implantation. Arrows point to cecum and the newly formed tumor in the cecum. (B) Colon to lung tumor metastasis was examined after four weeks implantation by H&E staining of lung tissue sections. Compared to clear lung tissue (left), a small tumor lesion was observed in the lung (middle) from the HCT116 p53−/− implantation group; whereas large and multiple colon tumor lesions were found in the NANOG-HCT116 p53−/− implantation group (right). (C) Statistical comparison of lung metastasis frequency in the orthotopic cecum implantation model derived from HCT116 p53−/− (n = 8) and NANOG-HCT116 p53−/− (n = 11). (D) Flow cytometry analysis of CD133 expression in HCT116 cells demonstrated three populations of cells; namely CD133−, CD133+^low^, and CD133+^high^. Less than 1% of cells were regarded as CD133+^high^. (E) *NANOG* promoter methylation patterns in CD133−, CD133+^low^, and CD133+^high^ HCT116 cells. CD133+^high^ cells demonstrated a significant reduction of *NANOG* promoter methylation. (F) *NANOG* expression in CD133−, CD133+^low^, and CD133+^ high^ HCT116 cells. CD133+^high^ cells demonstrated a significantly increased *NANOG* expression. The *NANOG* expression in CD133− was defined as 1, and fold increase in CD133+^low^ and CD133+^ high^ (versus to CD133−) was calculated as ΔΔCT. Data are the mean ± SD from three experiments with duplicates. (G) HCT116 cells were infected with *NANOG*-GFP lentivirus and analyzed for CD133 populations by flow cytometry. The percentages of CD133 populations were compared between HCT116 cells with either endogenous level or overexpression level of NANOG. The CD133+^high^ population (R5) was increased in cells with overexpression of *NANOG*.

Following the identification of *NANOG* upregulation in metastatic HCC cells and tumor samples, we furthered our study to examine the *in vivo* function of *NANOG* in cancer progression. However, the strong expression of *NANOG* in HCC cells makes it difficult to be knocked down efficiently for *in vivo* functional studies (data not shown). We, therefore, selected the HCT116 cell line, which has low endogenous level of NANOG, for overexpression study. A stable *NANOG* expressing HCT116 p53−/− cells (Figure S6A in File S1) were injected into nude mice for tumor formation. Surprisingly, a minimum of 1×10^4^ cells of either HCT116 p53−/− or *NANOG* expressing HCT116 p53−/− (NANOG-HCT116 p53−/−) cells was sufficient to form subcutaneous tumors (Table S4), indicating that a higher level of *NANOG* expression could not further enhance the formation of primary tumors. However, using an orthotopic tumor implantation mouse model, we observed a significantly higher colon to lung metastasis by implanting xenograft tumors of NANOG-HCT116 p53−/− cells compared to the parental HCT116 p53−/− cells (Figure 4A–C). This provides strong *in vivo* evidence to support the function of *NANOG* in promoting cancer cell metastasis.

### Demethylation of NANOG in CD133+^high^ population of HCT116 cells

High expression of CD133 is one of the indications of CSC population in various types of cancers, including colon cancer [21], [22]. Since the three pluripotent genes *NANOG*, *OCT4*, and *c*-*MYC* were hypermethylated in colorectal carcinoma HCT116 cells, we asked whether the same pattern of methylation would be observed in the rare putative CSC population in HCT116 cells. We observed over 80% of HCT116 cells were CD133+, but only 1% of cells were regarded as CD133+^high^ (Figure 4D). Importantly, *NANOG* promoter methylation was maintained at high level in both CD133− and CD133+^low^ HCT116 cells (89% and 78%, respectively), while it was dramatically reduced to 42% in CD133+^high^ cells (Figure 4E). The reduced methylation was also found in accordance with a significant upregulation of *NANOG* expression in CD133+^high^ cells (Figure 4F). In addition, overexpression of exogenous *NANOG* in HCT116 cells resulted in an increase of CD133+^high^ population (Figure 4G), suggesting that demethylation of *NANOG* promoter contributes to the initiation of CSCs in tumor development.

### Pluripotency regulatory circuits in cancer cells

**Figure 5:**
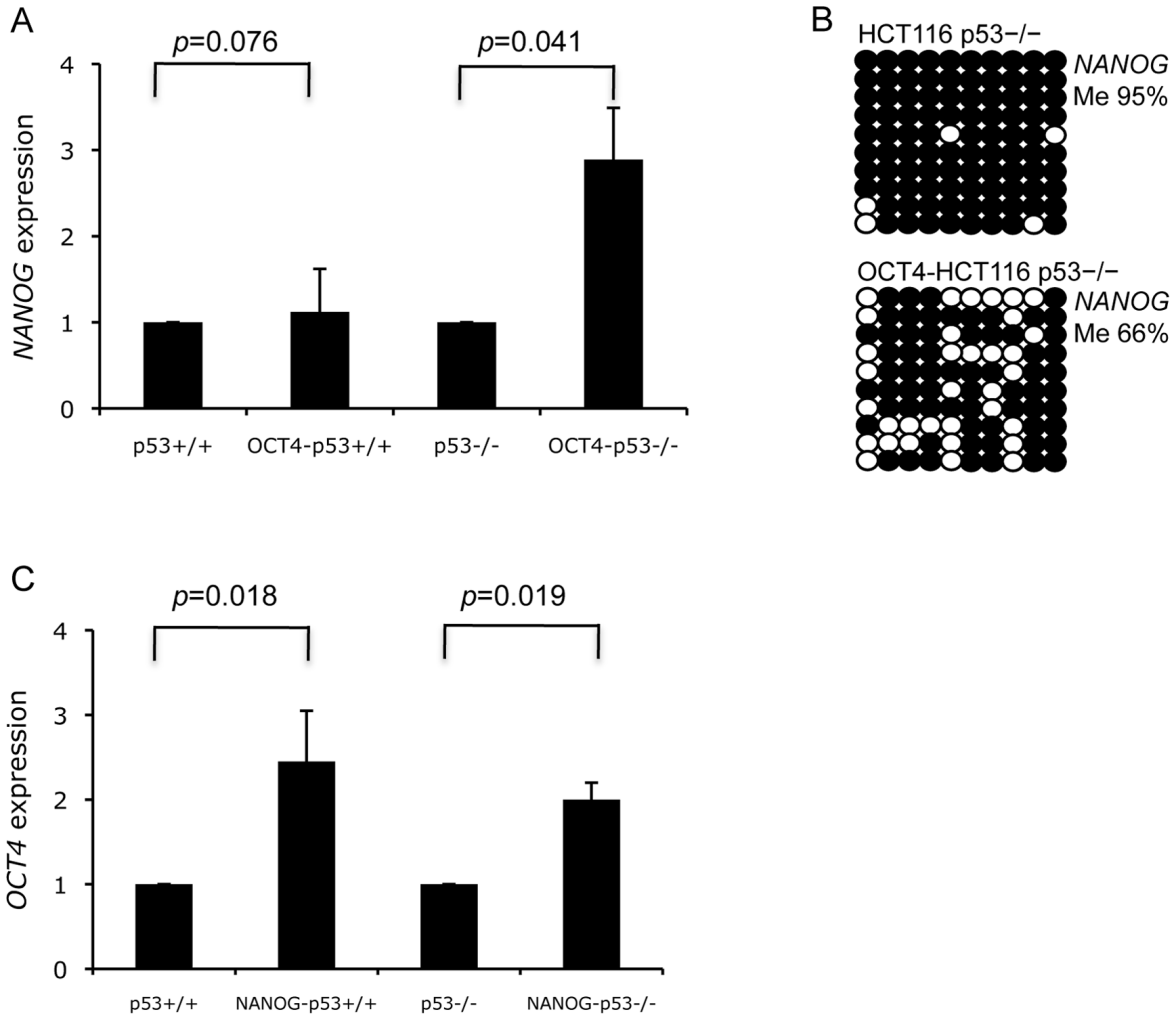
Cross-regulation of NANOG and OCT4 in HCT116 cancer cells. (A) HCT116 p53+/+ and p53−/− cells were infected with *OCT4*-GFP-lentivirus. Strong induction of endogenous *NANOG* expression was found in OCT4-expressing HCT116 p53−/− cells. The gene expression in HCT116 cells without infection was defined as 1, and fold increase in HCT116 cells with infection was calculated by ΔCT. Data are the mean ± SD from three experiments with duplicates. Paired Student *t* test was used for statistical analysis. (B) *NANOG* promoter methylation patterns in HCT116 p53–/– cells with or without *OCT4* overexpression. (C) HCT116 cells were infected with *NANOG*-GFP-lentivirus. Significant induction of endogenous *OCT4* expression was found in both NANOG-expressing HCT116 p53+/+ and p53−/− cells. The gene expression calculation was the same as described in (A). Paired Student *t* test was used for statistical analysis.

*OCT4*, *SOX2*, and *NANOG* have been demonstrated to form a transcriptional regulatory loop in ESCs for the maintenance of pluripotency [23]–[25]. We asked whether such cross-regulation is conserved in cancer cells with aberrant epigenetic patterns. HCT116 cells with different p53 genetic backgrounds were used because p53 was reported as a barrier to the re-establishment of pluripotency [26]. Interestingly, overexpression of exogenous *OCT4* significantly increased *NANOG* mRNA levels in HCT116 p53−/− cells, but not in HCT116 p53+/+ cells (Figure 5A; Figure S6B in File S1). The induction of *NANOG* expression was associated with a decreased promoter methylation in OCT4-HCT116 p53−/− cells (from 95% to 66%; Figure 5B). Moreover, overexpression of the exogenous *NANOG* significantly enhanced *OCT4* mRNA levels in HCT116 cells regardless of their p53 status (Figure 5C). This suggests that the pluripotency regulatory circuit is conserved in cancer cells and it is partially restored in cancer cells via the epigenetic mechanism that reprograms *NANOG* promoter methylation.

## Discussion

Activation of the molecular targets of pluripotency-associated genes (*NANOG*, *OCT4*, *SOX2*, and *c*-*MYC*) is frequently observed in poorly differentiated cancers [11], [27]. *OCT4* and *NANOG* expression have also been found associated with CSC properties in human cancers [28]–[33]. In this study, we have demonstrated a stem cell epigenetic signature of the pluripotency-associated genes in cancer cell lines; in particular an increased expression of *NANOG* in metastatic HCC cells and in poorly prognostic human HCC tumors (Figure 1B, 1C, 2A, 3; Table 1), which presumably is associated with its promoter hypomethylated status and the suppression of *p53*. We also noticed that *NANOGP8*, encodes a protein with over 99.5% similarity to the authentic NANOG1 protein, is expressed in a variety of cancer cells, including HCT116 cells [34], [35]. It is possible that *NANOGP8* level may interfere with the detection of *NANOG* expression in our study since the TaqMan probe used cannot distinguish between the two gene sequences. Importantly, our data indicated that the strong *NANOG* expression was associated with the initiation of a putative CSC population (Figure 4D–G) and promoted *in vivo* tumor metastasis (Figure 4A–C). Therefore, we hypothesize that demethylation of *NANOG* occurs during tumor development and the corresponding expression of *NANOG* provides tumor cells with metastatic CSC properties.

Cancer cells have been proposed to adapt to an epigenetic signature of “stemness”. Different cancer cells display their unique patterns of DNA methylation, histone modifications, and pluripotent gene expression that may reflect the different tissue origins. Interestingly, iPSCs harbour residual DNA methylation signatures of their somatic tissue origin [13]–[16]. Although it is yet to be determined whether the phenomenon of epigenetic memory associates with cancer development, our data suggest the possibility that different tissue origins of cancers might hold different carcinogenic potentials when they are subjected to epigenetic alterations, of which some are more prone to re-activate pluripotent genes by acquiring a stem cell epigenetic signature.

OCT4 and NANOG are the core components of protein complexes for maintaining pluripotency and activating transcriptional regulatory circuit. Our observation of *OCT4*/*NANOG* cross-induction in HCT116 cells further demonstrates a conservation of cross-regulatory function between pluripotent factors in cancer cells (Figure 5). Apparently this re-activation of transcriptional circuits depends on epigenetic reprogramming of the silent promoters in cancer cells. It is noted that the suppressive role of p53 in stem cell pluripotency is supported by iPSC studies [36], [26], [37]. In our study, the *NANOG* promoter was hypermethylated in both HCT116 p53+/+ and p53−/− cells (Figure 4E and 5B), suggesting that depletion of p53 function alone does not affect the ground state of cancer epigenetic patterns. However, enforced expression of OCT4 caused a partial demethylation of the *NANOG* promoter in p53-null cells, but not in p53-expressing cells (Figure 5B), suggesting that inactivation of p53 facilitates epigenetic reprogramming of pluripotency circuits in cancer cells and aids in conferring them with CSC properties.

In conclusion, our results demonstrate that epigenetic regulation is important for the expression of pluripotency-associated genes in cancer cells, particularly *NANOG*, and contributes to the metastatic potential of CSCs. We believe that the re-activation of pluripotency circuits by aberrant epigenetic alterations is one of the key events of CSC initiation.

## Supporting Information

File S1 **Figure S1, Diagram of** ***KLF4*** **and** ***SOX2*** **regulatory regions, with location of bisulfite sequencing (BiS) primers (red lines) and ChIP primers (green line).** (A) The *KLF4* promoter covers 49 CpG sites from −388 to +52. (B) The *SOX2* promoter covers the distal region of 10 CpG sites (−1502 to −1373) and the proximal region of 19 CpG sites (−175 to −18). **Figure S2,** ***c-MYC*** **methylation patterns at the promoter region, before TSS1 and TSS2, and at exon 2.** *c-MYC* methylation patterns and frequency (%) at the indicated promoter region: (A) in normal PBMC; (B) in 97L cells; (C) in HCT116 cells; and (D) at exon 2 in PBMC, MIHA, PLC, and 97L cells, respectively. Open circles represent unmethylated CpGs; closed circles represent methylated CpGs. **Figure S3**, *KLF4* methylation patterns and frequency (%) at the promoter region before the TSS: (A) in PBMC; (B) in PLC; and (C) in HCT116 cells. **Figure S4**, *SOX2* methylation patterns and frequency (%) at the promoter region before the TSS: (A) in PBMC; (B) in MIHA; (C) in PLC; and (D) in 97L cells. **Figure S5**, Expression levels of: (A) *NANOG*; (B) *OCT4*; (C) *c*-*MYC*; and (D) *KLF4* genes were determined in L02, PLC, 97L, and HCT116 cells by qRT-PCR analysis normalized with the reference gene *β-ACTIN*. Data are the mean ± SD obtained from 2 to 3 experiments with duplicates. **Figure S6, Overexpression of exogenous** ***NANOG*** **and** ***OCT4*** **, facilitated by lentivirus infection.** (A) Overexpression of the exogenous *NANOG* gene was detected in HCT116 p53+/+ and p53−/− cells which were infected with NANOG-GFP-lentivirus. (B) Overexpression of the exogenous *OCT4* gene was detected in HCT116 p53+/+ and HCT116 p53−/− cells which were infected with OCT4-GFP-lentivirus.
(PDF)

Table S1 **Primers for bisulfite sequencing and ChIP-qPCR.**
(DOCX)

Table S2 **Primers for qPCR.**
(DOCX)

Table S3 ***NANOG*** **methylation and expression, and** ***p53*** **expression in HCC.**
(DOCX)

Table S4 **Tumor formation in HCT116 cells with or without exogenous NANOG overexpression.**
(DOCX)
