# Supplementary figures and images for: Epigenetic Regulation of Pluripotent Genes Mediates Stem Cell Features in Human Hepatocellular Carcinoma and Cancer Cell Lines
Source: PLoS One. 2013 Sep 4;8(9):e72435. doi: 10.1371/journal.pone.0072435 (PMC3762826; doi:10.1371/journal.pone.0072435)

## Supporting figures

### Figure S1

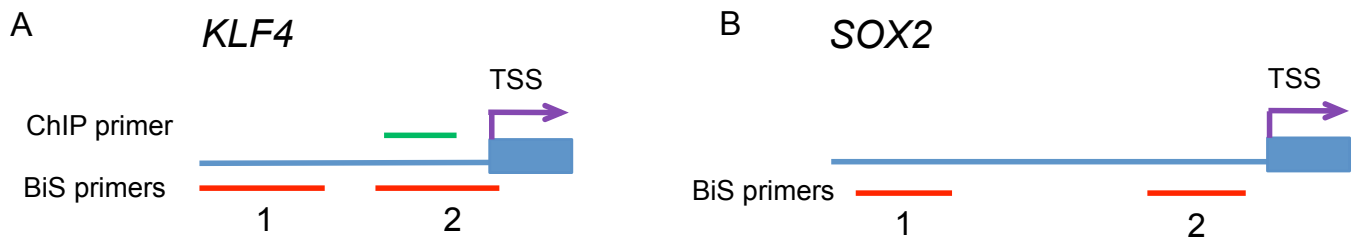

### Figure S2

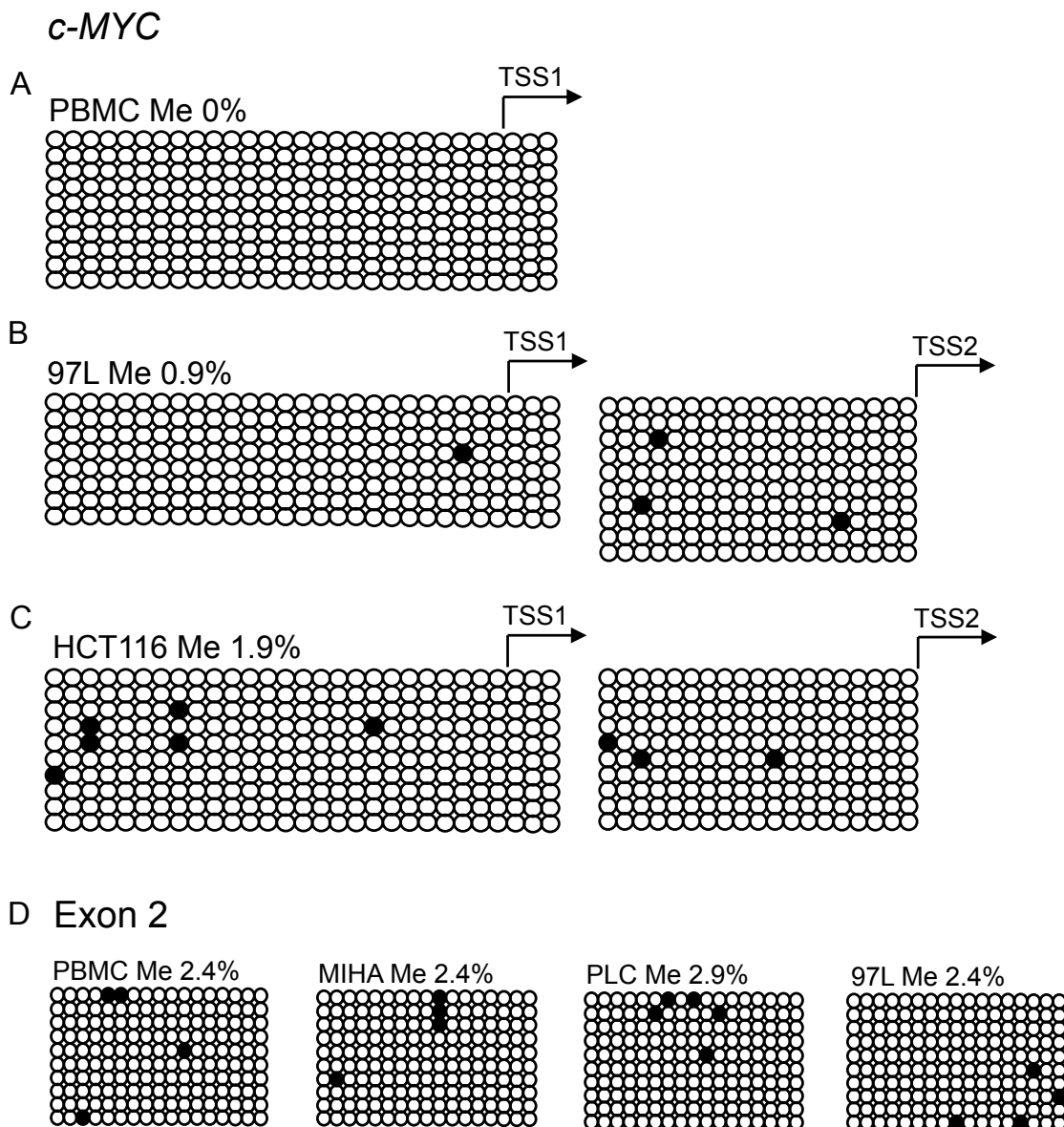

**Figure S3**

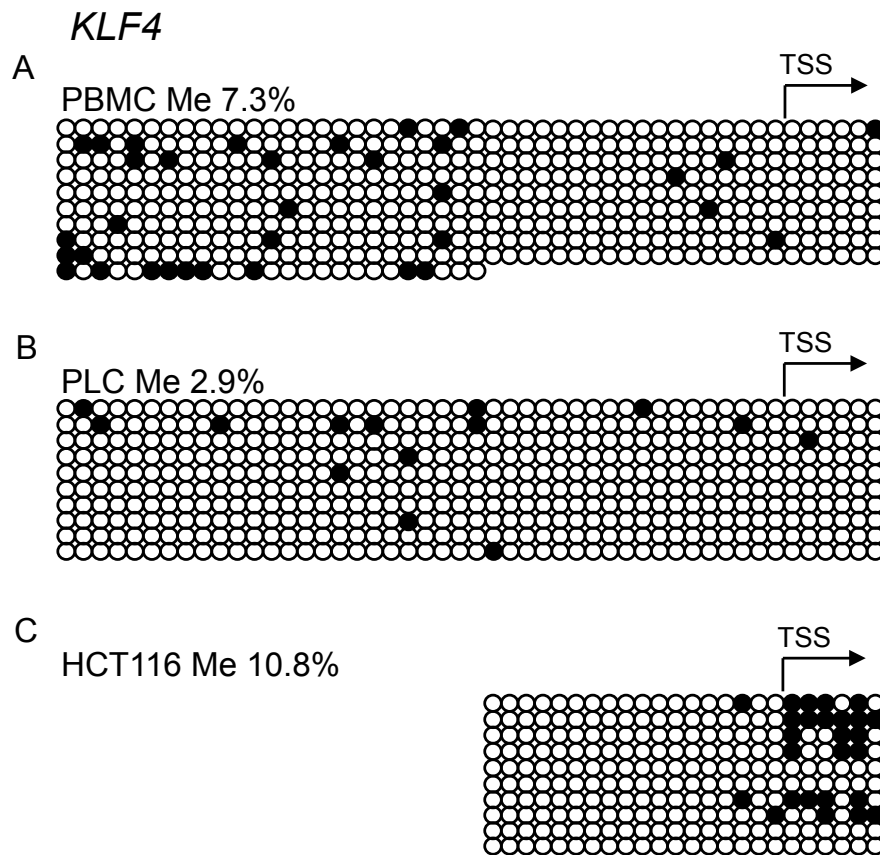

**Figure S4**

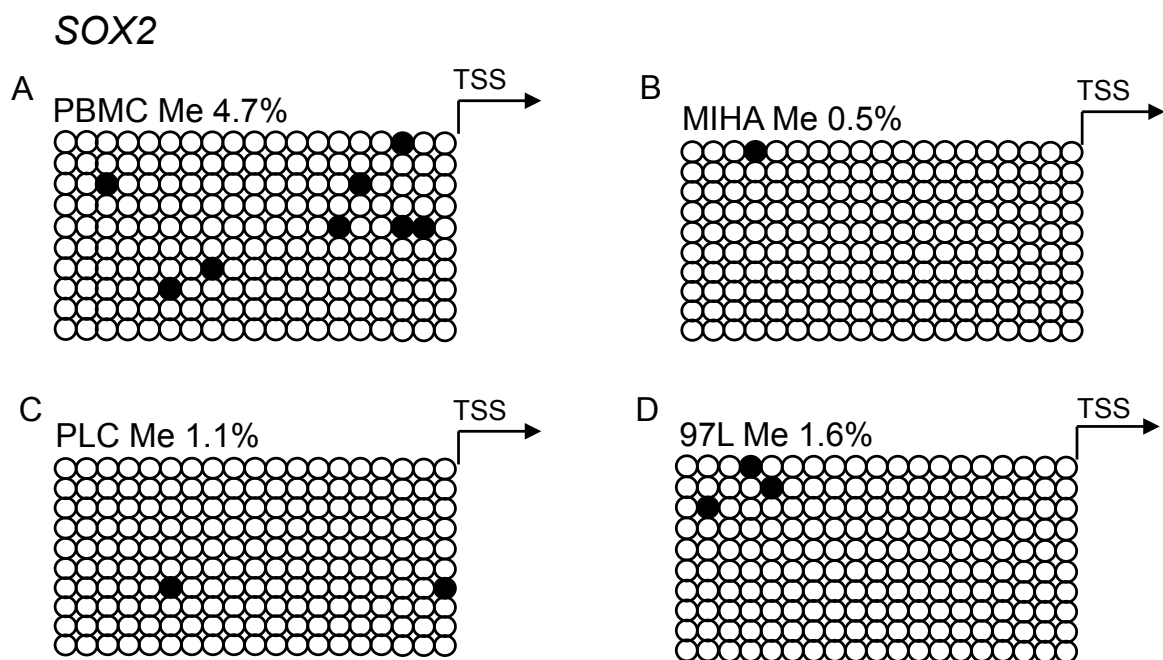

**Figure S5**

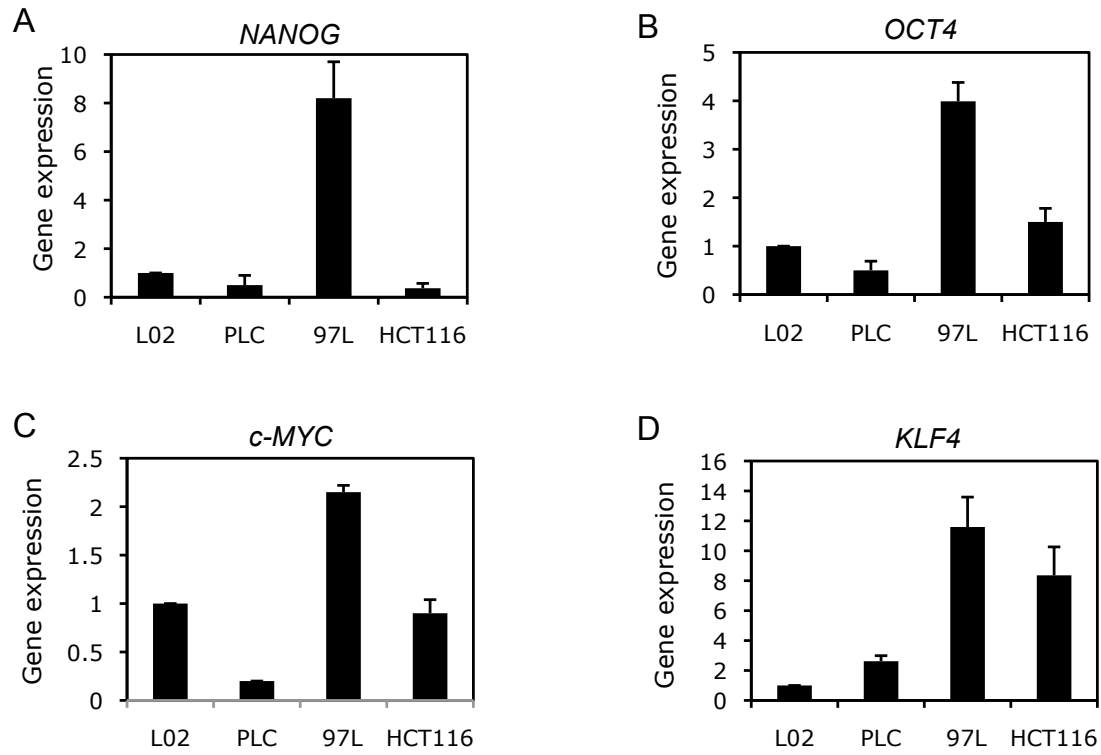

**Figure S6**

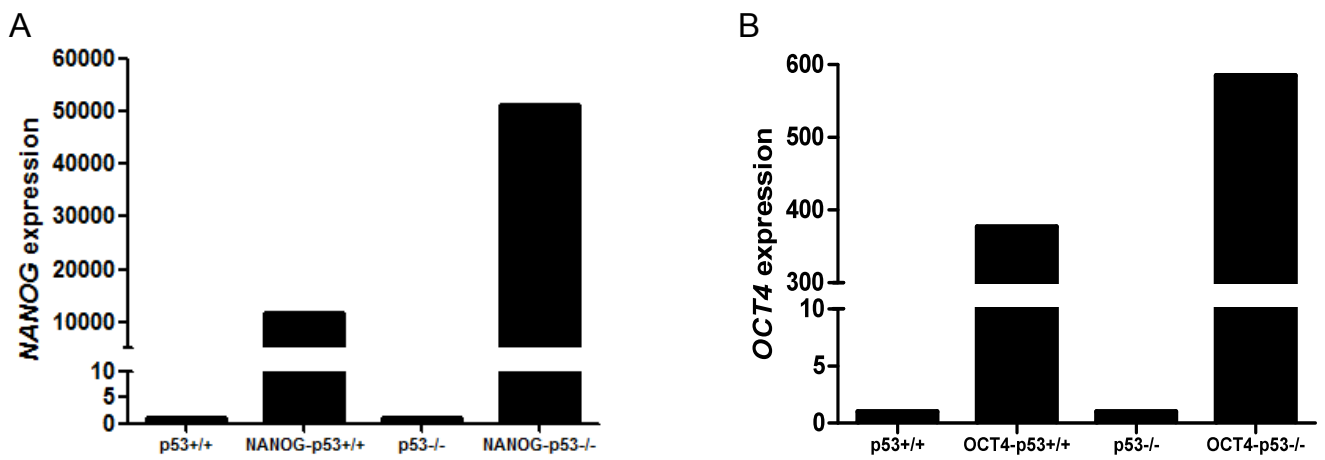

Supplement: File S1 — Figure S1, Diagram of KLF4 and SOX2 regulatory regions, with location of bisulfite sequencing (BiS) primers (red lines) and ChIP primers (green line). (A) The KLF4 promoter covers 49 CpG sites from −388 to +52. (B) The SOX2 promoter covers the distal region of 10 CpG sites (−1502 to −1373) and the proximal region of 19 CpG sites (−175 to −18). Figure S2, c-MYC methylation patterns at the promoter region, before TSS1 and TSS2, and at exon 2. c-MYC methylation patterns and frequency (%) at the indicated promoter region: (A) in normal PBMC; (B) in 97L cells; (C) in HCT116 cells; and (D) at exon 2 in PBMC, MIHA, PLC, and 97L cells, respectively. Open circles represent unmethylated CpGs; closed circles represent methylated CpGs. Figure S3, KLF4 methylation patterns and frequency (%) at the promoter region before the TSS: (A) in PBMC; (B) in PLC; and (C) in HCT116 cells. Figure S4, SOX2 methylation patterns and frequency (%) at the promoter region before the TSS: (A) in PBMC; (B) in MIHA; (C) in PLC; and (D) in 97L cells. Figure S5, Expression levels of: (A) NANOG; (B) OCT4; (C) c-MYC; and (D) KLF4 genes were determined in L02, PLC, 97L, and HCT116 cells by qRT-PCR analysis normalized with the reference gene β-ACTIN. Data are the mean ± SD obtained from 2 to 3 experiments with duplicates. Figure S6, Overexpression of exogenous NANOG and OCT4 , facilitated by lentivirus infection. (A) Overexpression of the exogenous NANOG gene was detected in HCT116 p53+/+ and p53−/− cells which were infected with NANOG-GFP-lentivirus. (B) Overexpression of the exogenous OCT4 gene was detected in HCT116 p53+/+ and HCT116 p53−/− cells which were infected with OCT4-GFP-lentivirus. (PDF) [file pone.0072435.s001.pdf]
