# Supplementary material for: Epigenetic Regulation of Pluripotent Genes Mediates Stem Cell Features in Human Hepatocellular Carcinoma and Cancer Cell Lines
Source: PLoS One. 2013 Sep 4;8(9):e72435. doi: 10.1371/journal.pone.0072435 (PMC3762826; doi:10.1371/journal.pone.0072435)
Supplement: Table S1 — Primers for bisulfite sequencing and ChIP-qPCR. (DOCX) [file pone.0072435.s002.docx]

Supporting tables

Table S1. Primers for bisulfite sequencing and ChIP-qPCR

| Genes | Primer pair | Forward primer (F)  Reverse primer (R) | Covered Seq. relative to TSS |
| --- | --- | --- | --- |
| **Bisulfite sequencing primers** | | | |
| *NANOG*  *OCT4*  *c-MYC*  *KLF4*  *SOX2* | Nanog-1  Nanog-2  Oct4-1  Oct4-2  Oct4-3  Oct4-4  Oct4-5  Oct4-6  Oct4-7  Oct4-8  Myc-1  Myc-2  Myc-3  Myc-4  Myc-5  Myc-6  Klf-1  Klf-2  Sox-1  Sox-2 | F: GATAGGAGGGTAAGTTTTTTTTT  R: CCAAAACAACTAACTTTACT  F: GAGTTAAAGAGTTTTGTTTTTAAAAATTAT  R: TCCCAAATCTAATAATTTATCATATCTTTC  F: TTTTTAGTTTTTTTTAGGTTTAA  R: CTAAACAAAAAACCCATTCC  F: TGGGTAGTAGGGATTTTTTGGATTG  R: TTTTAAAACTTTTCCCCCACTCTTATATT  F: GGGGAAAAGTTTTAAAAGATTTA  R: ATTCTATTTACAAAAAAATAACCAA  F: GGGAGTTTAGGGTAGTTTTTTTGTA  R: AAACTACTTCCTACTCCCCAACC  F: GGTGTGGGAGTGATTTTAGATAGTT  R: ATCTTAAATTCCTATCCTCAAAAAATC  F: GAAGGATTGTTTTGGTTTAGTAGTAGAT  R: CACCCACTAACCTTAACCTCTAAC  F: GGGGTTAGAGGTTAAGGTTAGTG  R: ATCACCTCCACCACCTAAAAAA  F: TTTTTAGGTGGTGGAGGTGAT  R: CCCTCAAACTAAAAAATCTCCAAAC  F: TTTTATTTGTTTTTTTTAGGAAGTT  R: TACTCTCTACCAATCTATACCCCAC  F: AAATTTGGGTTTTTAGAGGTGTTAG  R: CCCCACCAAAAATAACAACTATTC  F: ATAGTTGTTATTTTTGGTGGGGT  R: TTTACCAACTTTTCTTCTTTCTCTC  F: AATTTTGTGTTTTGGATTTTGGTAA  R: CCCTTTAAACAATCCCCTACTATCT  F: TTGGGAGGAGATATGGTGAATTA  R: CAAATACAAACTAAAAATAAAACAAAC  F: GGAAGGATTATTTTGTTGTTAAGAGG  R: AAACCTTTTCATTATTTTCCAACTC  F: TTTGGGGTTATTAAGAGTTATTGAA  R: AAACTCACTCTAACCCAACCAATAT  F: TTTAGATATTGGTTGGGTTAGAGTGA  R: AAAACTAATCAAACAAAAAACACCC  F: TTAATAAGAGAGTGGAAGGAAATTTAGA  R: AAAACCCAAAAAAATAATTTTAACC  F: GTAGGTTGGTTTTGGGAGTTTTT  R: AAATTAATAAACAACCATCCATATAAC | -1449 to -1295  -1168 to -952  -2973 to -2724  -2564 to -2184  -2004 to -1836  -1717 to -1479  -530 to -464  -301 to -58  +7 to +91  +130 to +320  -2620 to -2435  -348 to -164  -126 to +18  +289 to +438  Exon 2  Exon 3  -388 to -196  -160 to +52  -1502 to -1373  -175 to -18 |
| **ChIP qPCR primers** | | | |
| *NANOG*  *OCT4*  *c-MYC*  *KLF4* | Nanog  Oct4  c-Myc  Klf4 | F: TGAATGTTGGGTTTGGGAAT  R: GCTTTTTCCCTCTGGCTCTT  F: AGTCTGGGCAACAAAGTGAGA*  R: AGAAACTGAGGCGAAGGATG*  F: CAGATCAGCAACAACCGAAA  R: GGCCTTTTCATTGTTTTCCA  F: TGGGCCAGAGTGAGTTTAGC  R: GCGGGTGTTATGTCCTGTCT | -1101 to -883  -262 to -94  Exon 3  -179 to -20 |

* From Freberg CT, Dahl JA, Timoskainen S, Collas P. Mol Biol Cell 2007;18(5):1543-1553.
