# Supplementary material for: Epigenetic Regulation of Pluripotent Genes Mediates Stem Cell Features in Human Hepatocellular Carcinoma and Cancer Cell Lines
Source: PLoS One. 2013 Sep 4;8(9):e72435. doi: 10.1371/journal.pone.0072435 (PMC3762826; doi:10.1371/journal.pone.0072435)
Supplement: Table S2 — Primers for qPCR. (DOCX) [file pone.0072435.s003.docx]

Supporting tables

Table S2. Primers for qPCR

| Genes | Forward primer (F)  Reverse primer (R) |
| --- | --- |
| *OCT4* | F: CTCACCCTGGGGGTTCTATT  R: CTCCAGGTTGCCTCTCACTC |
| *KLF4* | F: CCCACACAGGTGAGAAACCT  R: ATGTGTAAGGCGAGGTGGTC |
| *c-MYC* | F: GGCTCCTGGCAAAAGGTCA  R: CTGCGTAGTTGTGCTGATGT |
| *p53* | F: GTTCCGAGAGCTGAATGAGG  R: TCTGAGTCAGGCCCTTCTGT |
| *β-ACTIN* | F: GGACTTCGAGCAAGAGATGG  R: AGCACTGTGTTGGCGTACAG |
