# Supplementary material for: Epigenetic Regulation of Pluripotent Genes Mediates Stem Cell Features in Human Hepatocellular Carcinoma and Cancer Cell Lines
Source: PLoS One. 2013 Sep 4;8(9):e72435. doi: 10.1371/journal.pone.0072435 (PMC3762826; doi:10.1371/journal.pone.0072435)
Supplement: Table S3 — NANOG methylation and expression, and p53 expression in HCC. (DOCX) [file pone.0072435.s004.docx]

Supporting tables

Table S3. *NANOG* methylation and expression, and *p53* expression in HCC tumor samples

|  |  |  |  |  |
| --- | --- | --- | --- | --- |
| TNM stage | Patients # | *NANOG* methylation (%) | *NANOG* expression (2^∆∆Ct^)* | *p53* expression  (2^∆∆Ct^)* |
|  |  |  |  |  |
| I | 1140 | 45 | 0.83 | 0.8 |
|  | 1263 | 19 | 1.04 | 1.4 |
| II | 411 | 20 | 1.24 | 1.67 |
|  | 245 | 29 | 1.38 | 3.2 |
|  | 1177 | 20 | 0.55 | 0.5 |
|  | 1731 | 26 | 3.22 | 1.66 |
| III | 23 | 27 | 1.5 | 0.9 |
|  | 392 | 36 | 8.44 | 0.44 |
|  | 812 | 51 | 5.49 | 0.4 |
|  | 1129 | 22 | 1.82 | 0.71 |
|  | 1391 | 34 | 4.64 | 2.25 |
| IV | 297 | 23 | 3.23 | 0.57 |
|  | 705 | 30 | 1.8 | 0.13 |
|  | 975 | 36 | 0.97 | 0.63 |
|  | 994 | 32 | 1.51 | 0.4 |

**NANOG* and *p53* expression levels in non-tumor tissue was defined as 1, and fold

change in tumor tissue (versus non-tumor tissue) was calculated by ΔΔCT.
