# Supplementary material for: Epigenetic Regulation of Pluripotent Genes Mediates Stem Cell Features in Human Hepatocellular Carcinoma and Cancer Cell Lines
Source: PLoS One. 2013 Sep 4;8(9):e72435. doi: 10.1371/journal.pone.0072435 (PMC3762826; doi:10.1371/journal.pone.0072435)
Supplement: Table S4 — Tumor formation in HCT116 cells with or without exogenous NANOG overexpression. (DOCX) [file pone.0072435.s005.docx]

Supporting tables

Table S4. Tumor formation in HCT116 cells with or without exogenous

NANOG overexpression

| Cell type | Cell injection | Tumor formation | *p* |
| --- | --- | --- | --- |
| HCT116 p53+/+ | 1 x 10^4^ | 57% (4/7) |  |
| NANOG-HCT116 p53+/+ | 1 x 10^4^ | 78% (7/9) | 0.426 |
| HCT116 p53−/− | 1 x 10^4^ | 100% (4/4) |  |
| NANOG-HCT116 p53−/− | 1 x 10^4^ | 100% (6/6) | NT |
